# Immunotherapy of thymic epithelial tumors: molecular understandings and clinical perspectives

**DOI:** 10.1186/s12943-023-01772-4

**Published:** 2023-04-13

**Authors:** Yong-Qiang Ao, Jian Gao, Shuai Wang, Jia-Hao Jiang, Jie Deng, Hai-Kun Wang, Bei Xu, Jian-Yong Ding

**Affiliations:** 1 Department of Thoracic Surgery, Zhongshan Hospital, Fudan University, 180 Fenglin Road, 200032 Shanghai, China; 2 Cancer Center, Zhongshan Hospital, Fudan University, Shanghai, China; 3 Institute of Vascular Disease, Shanghai TCM-Integrated Hospital, Shanghai, China; 4 CAS Key Laboratory of Molecular Virology and Immunology, Institute Pasteur of Shanghai, Chinese Academy of Sciences, Shanghai, China; 5 Department of Oncology, Zhongshan Hospital, Fudan University, Shanghai, China

**Keywords:** Thymic epithelial tumors, Immunotherapy, Immune checkpoint inhibitors, PD-1, PD-L1, Immune-related adverse events

## Abstract

Immunotherapy has emerged to play a rapidly expanding role in the treatment of cancers. Currently, many clinical trials of therapeutic agents are on ongoing with majority of immune checkpoint inhibitors (ICIs) especially programmed death receptor 1 (PD-1) and its ligand 1 (PD-L1) inhibitors. PD-1 and PD-L1, two main immune checkpoints, are expressed at high levels in thymic epithelial tumors (TETs) and could be predictors of the progression and immunotherapeutic efficacy of TETs. However, despite inspiring efficacy reported in clinical trials and clinical practice, significantly higher incidence of immune-related adverse events (irAEs) than other tumors bring challenges to the administration of ICIs in TETs. To develop safe and effective immunotherapeutic patterns in TETs, understanding the clinical properties of patients, the cellular and molecular mechanisms of immunotherapy and irAEs occurrence are crucial. In this review, the progress of both basic and clinical research on immune checkpoints in TETs, the evidence of therapeutic efficacy and irAEs based on PD-1 /PD-L1 inhibitors in TETs treatment are discussed. Additionally, we highlighted the possible mechanisms underlying irAEs, prevention and management strategies, the insufficiency of current research and some worthy research insights. High PD-1/PD-L1 expression in TETs provides a rationale for ICI use. Completed clinical trials have shown an encouraging efficacy of ICIs, despite the high rate of irAEs. A deeper mechanism understanding at molecular level how ICIs function in TETs and why irAEs occur will help maximize the immunotherapeutic efficacy while minimizing irAEs risks in TET treatment to improve patient prognosis.

## Introduction

Thymic epithelial tumors (TETs) are the most common neoplasms of the anterior mediastinum in adults [1]. Histological classification includes thymomas (Type A, AB, B1, B2, B3), thymic carcinomas (Type C), and thymic neuroendocrine tumors, while Massaoka and Tumor-Node-Metastasis staging system concern localization of the involved areas [2]. TET treatment is a paradigm of multidisciplinary cooperation among surgeons, clinicians, and pathologists from establishing the diagnosis to determining therapeutic strategies, especially for complicated cases with autoimmune diseases [3]. Total thymectomy is currently the preferred strategy for TETs. For relapsed or refractory patients with local invasion or distant metastasis, cisplatin combined with anthracycline or paclitaxel has become the first choice [4]. However, the efficacy of chemotherapy is limited, and serious adverse reactions are observed. Targeted drugs such as receptor tyrosine kinase inhibitors and antiangiogenic agents are considered beneficial supplements after chemotherapy for advanced TET patients, but the efficacy is also not satisfactory. And a lack of utilizable genomic alterations in TETs hinders in the development of targeted therapies [5]. Immunotherapy plays an important role in tumor treatment, and the discovery of immune checkpoints drives tumor immunotherapy to a new stage [6]. Among all immune checkpoints, programmed death receptor 1 (PD-1) and its ligand 1 (PD-L1), and cytotoxic T lymphocyte-associated protein 4 (CTLA-4) have attracted increasing attention in TETs [7, 8]. CTLA-4 is expressed in TETs and positively correlates with a poor patient prognosis; moreover, it is associated with the pathogenesis of autoimmune diseases (ADs), such as myasthenia gravis [9]. Several clinical trials of CTLA-4 inhibitors in TETs are in progress, but the efficacy needs to be further examined in clinical practice of TET management. Accumulating evidence has confirmed high PD-1/PD-L1 expression in TETs that is associated with worse clinical characteristics and a poor patient prognosis [10], showing great immunotherapeutic potential. PD-1/PD-L1 inhibitors have been used to treat a variety of tumors, such as melanoma and non-small cell lung cancer (NSCLC), and have shown significant efficacy [11–13]. Many clinical trials evaluating the efficacy of PD-1/PD-L1 inhibitors against TETs have also been completed or are in progress. Completed clinical trials reported both encouraging therapeutic benefits and worrisome immune-related adverse events (irAEs), making immunotherapy for TETs highly controversial. The National Comprehensive Cancer Network Guidelines version 2.2022 only recommends pembrolizumab as a second-line therapeutic strategy for thymic carcinoma (TC) with vigilance to the high incidence of irAEs, and no other immune checkpoint inhibitors (ICIs) are recommended for TET treatment [14]. This review summarizes the current research progress in ICIs as TET treatments, highlighting irAEs, the potential mechanisms, and prevention strategies. In addition to the deficiencies of current research, some viewpoints that are worthy of further consideration are noted, which will help lay the foundation and identify directions for future research on ICIs in TETs, accumulating more evidence for clinical practice.

### Immunotherapeutic agents for TETs

**Fig. 1:**
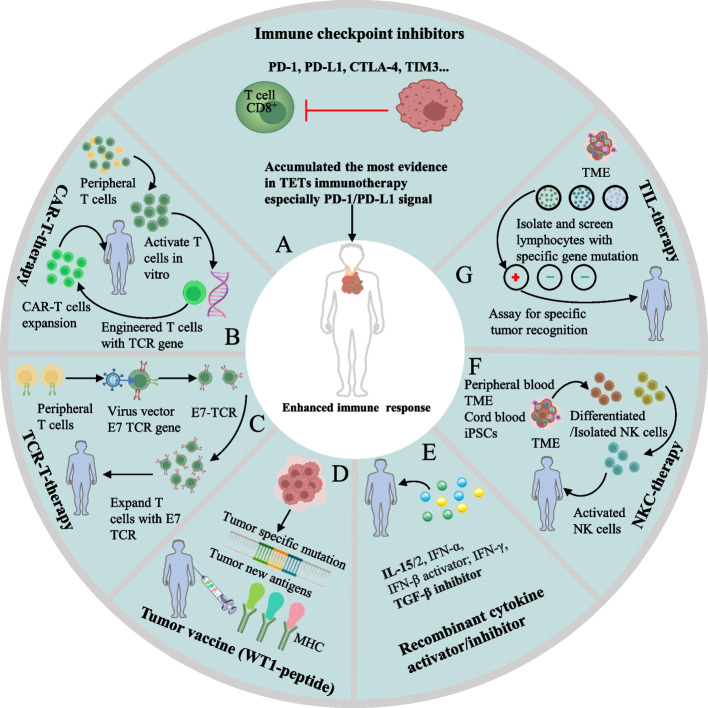
Different forms of anticancer immunotherapy. **A**. ICIs, especially PD-1/PD-L1 inhibitors, are the main immunotherapies used in TET treatment and have accumulated the most evidence. **B-E**. Adoptive cell therapies, including CAR-T, TIL, TCR-T and NKC therapies, and different sources of therapeutic cells are obtained, modified or screened and expanded for infusion back into the patients. **F**. Therapeutic vaccines are designed based on discovered tumor neoantigens. The infused tumor vaccines induce an immune response to tumor cells and enhance antitumor immunity. **G**. Cytokines with immune activation functions, such as IL-2 and IFN-α/β, are recombined and synthesized in vitro and then infused into patients, which enhance the antitumor immune response. CAR: Chimeric antigen receptor; TIL: Tumor-infiltrating lymphocyte; TCR: T cell receptor; NKC: Natural killer cell

The emergence and rapid advances in immunotherapies such as ICIs, cancer vaccines, cytokine-based therapies and adoptive cell therapies have significantly changed the treatment of cancers [15] (Fig. 1). They enhance antitumor immunity by blocking inhibitory signaling from immune checkpoints or by enhancing activity of stimulatory signaling, producing T cells with augmented responses toward tumor cells [16, 17]. Wilms’ tumor gene 1 (WT1), a small peptide overexpressed in TETs, can regulate cell reproduction and apoptosis, was reported to be involved in TETs pathogenesis. A phase II clinical trial that enrolled 18 patients with TETs was conducted to examine the efficacy of WT1-peptide vaccine [18] (Fig. 1. D). However, no patients achieved a complete or partial response, although 75% of patients had stable disease. Additionally, an in vivo experiment in mice [19] reported that adoptive transfer of B cells halts thymoma growth, which implies the potential of adoptive cell therapy in TETs. High expression of CD70, a protein belonging to the tumor necrosis family, was reported in TETs, and CD70-targeted CAR T cells were confirmed both in vitro and in vivo to be effective against tumors, indicating the possibility of CD70-targeted CAR T cell therapy for TETs [20–22]. Cytokines, such as interleukin (IL)-15 and interferon α can amplify patients’ antitumor immune responses [17], and activators of IL-15 in TETs are being explored in clinical trial (NCT04234113) (Fig. 1 E). Despite theoretically various immunotherapies for TETs, most of them are at experimental or preclinical level. The majority of evidence has been obtained from ICIs, especially PD-1/PD-L1 inhibitors (Fig. 1A), in both clinical trials and clinical practice of TET treatment.

### Rationale for using ICIs as TET treatments

#### The physiology and pathophysiology of the thymus

**Fig. 2:**
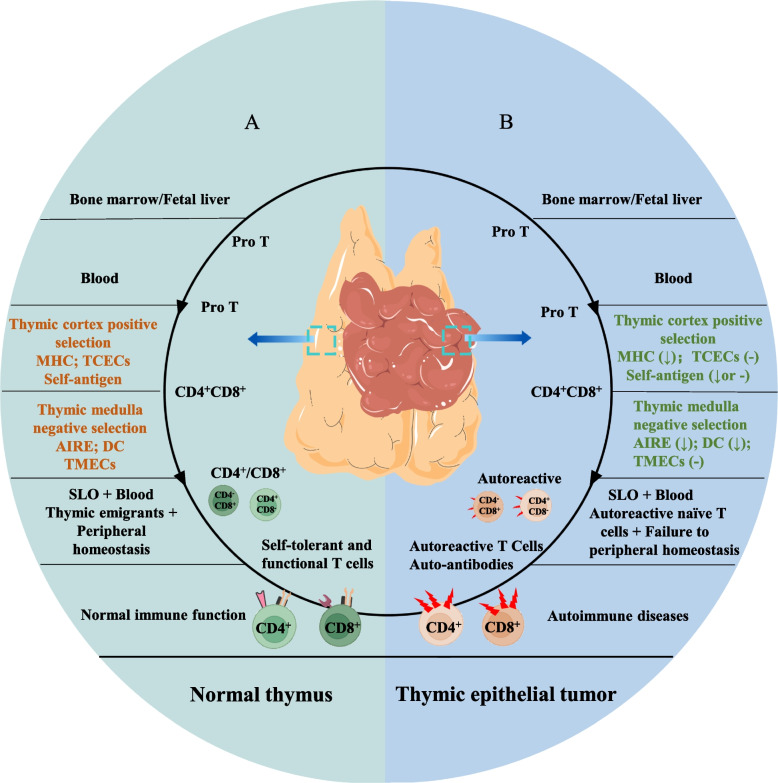
Naïve T cell development in the normal thymus and thymoma. **A**. A healthy thymus with a normal structure and thymic microenvironment determines normal T cell development and maturation and continuously exports normal naïve T cells to establish normal immune function. **B**. Thymic epithelial tumors with distorted structures and disrupted microenvironments lack components that are necessary for positive and negative selection. Naïve T cells do not complete the central immune tolerance and emigrate to become autoreactive RTEs, which directly or indirectly lead to autoimmune diseases through the cellular or humoral immune systems, respectively. Pro T: Progenitor T cells; AChR: Acetylcholine receptor; AIRE: Autoimmune regulator effectors; RTEs: Recent thymic emigrants; DC: Dendritic cells; SLO: Secondary lymphoid organs

Primitive progenitor T cells from the embryonic liver or bone marrow hematopoietic system migrate to the thymus, where they develop into naïve T cells that are immune-effective against allogeneic antigens and immune-tolerant to self-antigens after positive and negative selection [23]. Positive selection occurs in the thymic cortex by recognizing autogenic tissue-specific antigens (TSAs) presented by epithelial cells and major histocompatibility complex (MHC) molecules. This process allows CD4^−^CD8^−^ T cells to become CD4^+^CD8^+^ T cells, which also acquire T cell receptor (TCR) rearrangement and gain MHC restriction. Then, CD4^+^CD8^+^ T cells enter the thymic medulla, where they undergo negative selection mainly controlled by autoimmune regulator (AIRE) genes. Thymic medullary epithelial cells with high AIRE expression are prone to apoptosis and release TSAs that will be captured by dendritic cells and presented to CD4^+^CD8^+^ T cells through MHC. T cells that overreact undergo apoptosis, while surviving T cells recognize TSAs presented by MHC class I/II and develop into CD4^+^CD8^−^ or CD4^−^CD8^+^ T cells. This process is known as central immune tolerance, and these T cells subsequently egress to the peripheral circulation to become recent thymic emigrants, which plays an important role in building a complete immune system [23] (Fig. 2 A). In TETs, however, immune tolerance is rendered dysfunctional because of the decreased expression of AIRE, MHC and the altered thymic architecture. Consequently, an increased number of immature CD8^+^ T cells but decreased numbers of immature CD4^+^ T cells and regulatory T cells (Tregs) are observed [24]. These changes promote the release of autoreactive T cells, thus disturbing peripheral homeostasis, which, in turn, predisposes patients to autoimmunity or causes ADs (Fig. 2 B). Additionally, some cytokines, chemokines produced by tumor cells that induce cross-reactions between tumor antigens and TSAs, and the structural similarities between them, which promote autoantibody production, are other proposed mechanisms to explain autoimmunity in TETs [25, 26]. For instance, thymoma overexpresses mid-sized neurofilament gene, which shares sequences encoding acetylcholine receptors and titin epitopes and is often correlated with myasthenia gravis [26].

### PD-1 and PD-L1 are involved in T cell function

**Fig. 3:**
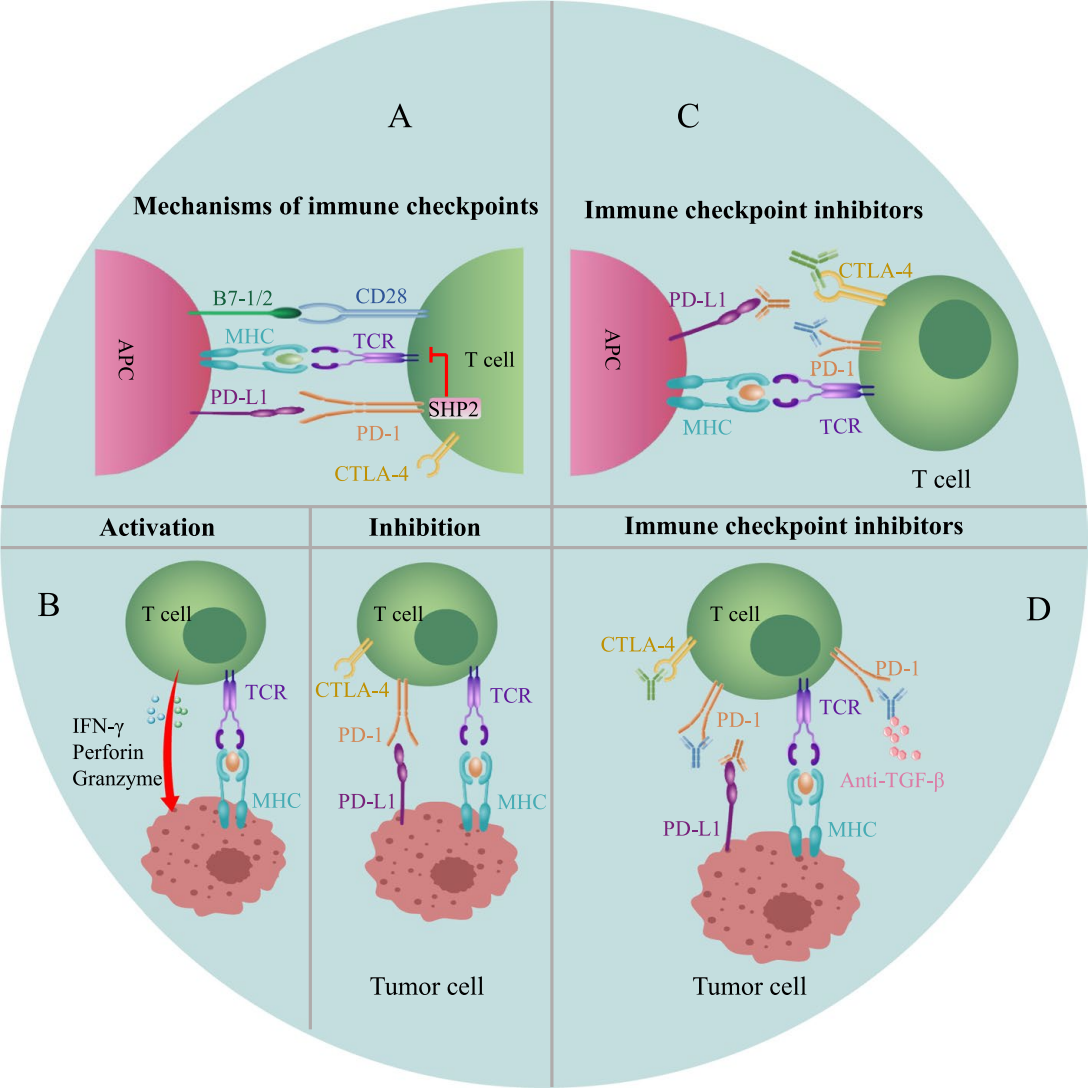
PD-1/PD-L1 signaling in tumor immune tolerance. **A**. The mechanism of PD-1/PD-L1-mediated inhibition of T cell activation and PD-1/PD-L1 blocker-mediated T cell function restoration (between APCs and T cells). **B**. The mechanism of PD-1/PD-L1-mediated tumor immune tolerance and PD-1/PD-L1 blocker-mediated antitumor activity. Blockade of PD-1/PD-L1 alone and in combination with anti-TGF-β restores immune-exhausted T cells. CTLA-4: Cytotoxic T lymphocyte-associated antigen 4; APC: Antigen-presenting cells; MHC: Major histocompatibility complex; TCR: T cell receptor; SHP2: Src homology 2 domain-containing tyrosine phosphatase 2

Activation of naïve T cells requires dual signals. The first is a specific antigen recognition signal that is acquired and processed by antigen-presenting cells and then recognized by T cells after an interaction with MHC molecules; the second is a costimulatory signal derived from B7-1/2 expressed by antigen-presenting cells and interacts with CD28 expressed on T cells [27] (Fig. 3 A). Only T cells exposed to both stimuli are activated, including tumor-infiltrating T cells [28]. Moreover, immunologists have discovered negative costimulatory signals, also known as immune checkpoints [6, 29, 30]. Under physiological conditions, immune checkpoints transmit signals that inhibit T cell activation, avoiding autoimmune responses, a process called peripheral immune tolerance. However, in tumor and inflammatory sites, immune checkpoints impair antitumor immunity and antiinfection ability, leading to chronic inflammation and tumor progression. Among all immune checkpoints, PD-1/PD-L1 represents the best studied checkpoint in TETs. PD-1 and PD-L1 are a receptor and ligand, respectively, mediating the cosuppressive signaling of T cells, immunosuppression of T cells and tumor immune escape (Fig. 3B). PD-1 is mainly expressed on immature CD4^–^/CD8^–^ thymocytes and activated CD4^+^/CD8^+^ T cells [31]. PD-L1 is constitutively expressed in different cells, such as dendritic cells, mediating peripheral immune tolerance [6]. However, PD-L1 is also expressed at high levels in inflammatory sites and tumor cells, impairing T cell-mediated immune function. PD-1 aggregates with TCR after binding to PD-L1 and associates with the Src homology 2 domain-containing tyrosine phosphatase 2. They compose negative costimulatory microclusters to induce the dephosphorylation of proximal TCR signaling molecules, inhibiting T cell activation [32] and inducing their differentiation into Tregs or apoptosis. Activation of PD-1/PD-L1 signaling has also been implicated in driving T cell exhaustion by limiting glucose and amino acid metabolism [33]. Therefore, blocking the PD-1/PD-L1 pathway may restore the antitumor effect of T cells (Fig. 3 C, D), which is also the rationale for using ICIs to treat tumors, including TETs that highly express PD-L1 [34].

### The clinical significance of PD-1/PD-L1 in TETs

TETs express PD-1 and PD-L1 at high levels ranging from 18–100% [7, 35–38] (Table 1), which differs between different Masaoka stages and TET subtypes, and usually a higher level is observed in thymic carcinoma (TC) than thymoma [39]. Possible explanations for significantly different expression levels include various detection methods and samples, different proportions of pathological subtypes, different diagnostic criteria for thymoma subtypes at different periods and different positive judgment criteria [40, 41]. In all, PD-L1 is expressed at high levels with a heterogeneous distribution among different subtypes of TETs. Additionally, PD-L1 has been revealed as a predictor of the response to TET immunotherapy. Through genomic and transcriptomic profiling of TET samples in the pembrolizumab treatment cohort, researchers found differentiate gene or molecular alterations associated with PD-L1 expression between responders and nonresponders [42, 43]. The relationship between PD-1/PD-L1 and the pathological characteristics or prognosis of patients with TETs is controversial; however, high PD-1/PD-L1 expression seems to indicate worse pathological characteristics and a poor patient prognosis in terms of both overall survival (OS) and progression-free survival (PFS) [44, 45]. Furthermore, PD-L1 expression is positively correlated with EMT-related indicators, which potentially predict the asymptomatic survival of patients [46].

**Table 1:** Expression level of PD-L1 in thymic epithelial tumors

No	Ref	TM/TC No	Antibody of PD-L1	Criteria of Positivity Judgement	Positivity rate
1	Katsuya et al. [47]	101/38	Clone E1L3N	Intensity of staining score (0–3) score 3	TM:23.0% TC: 70.0%
2	Padda et al. [48]	65/4	Clone15	Intensity of staining score (0–3) score 3	TM:68.0% TC: 75.0%
3	Marchevsky et al. [49]	38/8	SP142 (1:250)	Membranous expression ≥ 6%	TM:92.0% TC: 50.0%
4	Enkner et al. [50]	37/35	E1L3N	Staining H-score (cutoff value not defined)	TM:89.0% TC: 53.0%
5	Katsuya et al. [51]	12/18	E1L3N (1:800)	Intensity of staining score (0–3) score ≥ 1	TM:67.0% TC:41.0%
6	Yokoyama et al. [52]	82/0	EPR1161 (1:200)	Cutoff point of the PD-L1–positive rate was calculated to be 38% by Youden’s index	TM:53.7% TC: NA
7	Arbour et al. [36]	12/11	E1L3N	Membranous expression > 25%	TM: 92.0% TC: 36.0%
8	Tiseo et al. [53]	87/20	E1L3N (1:500)	Intensity of staining score (0–3) score 3	TM: 18.0% TC: 65.0%
9	Weissferdt et al. [35]	74/26	EPR4877 (1:250)	Membranous staining > 5%	TM: 64.0% TC: 54.0%
10	Suster et al. [37]	0/21	Clone SP142	Membranous staining > 50%	TM: NA TC: 71.4%
11	Owen et al. [54]	32/3	22C3	Intensity of staining (score 0–5) score 1	TM: 81.0% TC: 100%
12	Hakiri et al. [55]	81/0	SP142 (1:50)	Membranous expression ≥ 1%	TM: 27.0% TC: NA
13	Guleria et al. [56]	84/0	SP263	Membranous expression > 25%	TM: 82.0% TC: NA
14	Duan et al. [45]	20/13	Ab58810 (1:200)	Intensity of staining score (1–3). Median value of all scores as the cutoff value	TM: 65.0% TC: 46.2%
15	Chen et al. [57]	40/30	SP142	Membranous expression ≥ 5%	TM: 37.5% TC: 76.7%
16	Bagir et al. [58]	38/6	AM26531AF-N	Membranous staining > 5%	TM: 81.6% TC: 83.3%
17	Funaki et al. [46]	0/43	SP142(1:100)	Membranous staining > 50%	TM: NA TC: 60.5%
18	Wei et al. [44]	100/69	Clone E1L3N (1:100)	Membranous staining > 50%	TM:36% TC:37%
18	Higuchi et al. [38]	31/8	28–8	Membranous expression ≥ 1%	TM:51.6% TC:62.5%
19	Rouquette et al. [59]	53/50	Clone E1L3N Clone 22C3 Clone SP142 Clone SP263	Membranous staining > 1%	TM: > 92% to 98% TC: 66% to 73%
20	Ishihara et al. [60]	55/11	Clone SP263	Membranous staining > 25%	TM: 92.7% TC: 72.7%
21	Berardi et al. [10]	63/5	DAKO PD-L1 IHC 28–8 PharmDx kit	Membranous staining > 1%	Overall: 25%
22	Ishihara et al. [60]	66	Clone SP263 Clone AE1/AE3	The ratio of the PD-L1 positive rate to the AE1/AE3-positive rate	Overall: 38%

*TC* thymic carcinoma, *TM* thymoma, *Score 0–3* strong (3 +), *moderate (2* +*)* weak (1 +) or unstained (0), *NA* Not available, *IHC* immunohistochemistry

### Clinical exploration of PD-1/PD-L1 inhibitors in TET treatment

#### Completed clinical trials

PD-1/PD-L1 inhibitors have been shown to be effective antitumor agents and are approved for the treatment of various solid tumors by the Food and Drug Administration [11–13]. Clinical trials evaluating the efficacy of PD-1 antibodies (pembrolizumab and nivolumab) and a PD-L1 antibody (avelumab) against TETs have also accumulated some evidence. Four clinical trials have been completed and provide worthwhile information (Table 2), and the others are still in process (Table 3). It seemed that researchers considered the relatively better survival prognosis of TETs compared with other solid tumors, since most clinical trials adopt the overall/object remission rate (ORR) as the primary endpoint instead of PFS or OS.

**Table 2:** Completed clinical trials with PD-1/PD-L1 inhibitors in TETs

N	Intervention/Treatment	Phase	TC	Tm	DCR (%) (95%CI)	mPFS (95%CI)	mOS (months)	Primary endpoint	Identifier	Ref. & Year
1	Pembrolizumab	II	40	0	22.5(10.8–38.5)	4.2(2.9–10.3)	24.9	ORR	NCT02364076	Giaccone et al., 2018 [61]
2	Pembrolizumab	II	26	7	28.6/19.2	6.1(5.3–6.9)	14.5/NA	ORR	NCT02607631	Cho et al., 2019 [62]
3	Avelumab	I	1	7	57(NA)	NA	NA	DLT	NCT01772004	Rajan et al., 2019 [63]
4	Nivolumab	II	15	0	0(0–21.8)	3.8(1.9–7.0)	NA	ORR	PRIMER study NCCH1505	Katsuya et al., 2019 [64]

*N* number, *TC* thymic carcinoma, *Tm* Thymoma, *DCR* overall response rate, *mPFS* median recurrence free survival, *mOS* median overall survival, *ORR* Overall response rate, *DLT* Dose-limited toxicities

**Table 3:** Ongoing clinical trials with PD-1/PD-L1 inhibitors in TETs

N	Interventions	Phase	Patient(n)	Sponsors	Primary endpoint	Identifier
1	Nivolumab	II	55	European Organization for Research and Treatment of Cancer—EORTC	6 m-PFS	NCT03134118
2	Atezolizumab	II	34	Hoffmann-La Roche	ORR	NCT04321330
3	Nivolumab	II	117	Vanderbilt-Ingram Cancer Center	ORR	NCT03583086
4	Avelumab	II	55	National Cancer Institute (NCI)	ORR	NCT03076554
5	Chemotherapy + Pembrolizumab	IV	40	Tangdu Hospital, Fourth Military Medical University Xi'an, Shaanxi, China	ORR	NCT04554524
6	Pembrolizumab Lenvatinib 10 mg	II	43	Medica Scientia Innovation Research	5 m-PFS	NCT04710628
7	Toripalimab Chemotherapy	II	15	Shanghai Pulmonary Hospital, Shanghai, China	MPRR	NCT04667793
8	Pembrolizumab Radiation	II	40	Samsung Medical Center	MPRR	NCT03858582
9	KN046 (PD-L1& CTLA-4 inhibitor)	II	29	Weill Medical College of Cornell University	ORR	NCT04925947
10	KN046 (PD-L1& CTLA-4 inhibitor)	II	66	Jiangsu Alphamab Biopharmaceuticals Co., Ltd	ORR	NCT04469725
11	M7824 (PD-L1& TGF-ß inhibitor)	II	38	National Cancer Institute (NCI)	ORR	NCT04417660
12	SO-C101 (IL-15 activator) + Pembrolizumab	I	200	SOTIO Biotech AG	DLT	NCT04234113
13	XmAb20717 (PD-1 & CTLA-4 inhibitor)	I	154	Xencor, Inc	TRAE	NCT03517488
14	Pembrolizumab Sunitinib Malate (RTK inhibitor)	II	40	Ohio State University Comprehensive Cancer Center	ORR	NCT03463460

*PFS* Progression-free survival, *ORR* Objective response rate, *MPRR* Major pathologic response rate, *DLT* Dose-Limiting Toxicities, *TRAE* Treatment-related adverse events

## Pembrolizumab

Giaccone et al. [61] conducted a phase II clinical trial evaluating the efficacy of pembrolizumab in patients with recurrent TC. Patients with a history of ADs or immunodeficiency were excluded. Among 40 evaluable patients, an overall disease control rate of 22.5% was observed, with a disease progression rate of 25%. The median PFS of the PD-L1^high^ group was 4.2 months with an unachieved median OS, which was significantly higher than that of patients in the PD-L1^low^ group, with a median PFS of 2.9 months and a median OS of 15.5 months (*p* < 0.01), suggesting that the higher the PD-L1 expression level, the better the response to ICIs. However, irAEs were observed in all patients, in which 6 experienced more than one irAE and 6 had severe irAEs, such as myocarditis and hyperglycemia, but no deaths related to irAEs were reported. Giaccone et al. [65] updated the results of the long-term follow-up and reported that pembrolizumab induced durable responses in patients with TET, lasting approximately 3 years with a median survival of more than 2 years, and the 5-year survival rate was 18%. Notably, the incidence of severe irAEs (15%) did not increase significantly over time.

Cho et al. [62] included 33 patients with advanced TET in an open-label phase II clinical trial to evaluate the efficacy of pembrolizumab. Enrolled patients had received at least one first-line therapy, and those with a history of severe autoimmunity were excluded. Of seven patients with thymoma, two achieved partial responses, and five had stable diseases. Of the 26 patients with TC, five achieved partial responses, and 14 had stable diseases. The median duration of response was 9.7 months in patients with TC but was not reached in patients with thymoma. The median PFS was 6.1 months for both groups, and the median OS for patients with TC was 14.5 months but was not reached in patients with thymoma. Consistent with the results reported by Giaccone et al. [61], patients with higher PD-L1 expression had a better response to immunotherapy. Both Cho et al. [62] and Giaccone et al. [60, 61] demonstrated that pembrolizumab yielded encouraging antitumor activity with durable response in refractory, metastatic, or relapsed TETs in their clinical trials and updated follow-up data. They reported a significant correlation between high PD-L1 expression and better response to pembrolizumab in TETs and found that patients with durable responses had high PD-L1 expression. Some research reported durable response to immunotherapy in other solid tumors such as non-small cell lung cancer, breast cancer, and prostate cancer [66–69], and tumor mutational burden, microsatellite instability and high expression of immune checkpoint might contribute to this phenomenon. Some studies also demonstrated that TETs with microsatellite instability existed and might be sensitive to immunotherapy [70, 71]. Recently, Repetto et al. [72] reported a thymic carcinoma with Lynch syndrome, which had microsatellite instability and achieved durable response to avelumab and axitinib combination therapy. Collectively, TET patients with microsatellite instability and high expression of immune checkpoint might obtain durable response in immunotherapy.

## Avelumab

Rajan et al. [63] evaluated the efficacy of avelumab in 8 patients with TETs without a history of autoimmunity. Four of the seven patients with thymoma responded, and the other three had stable disease. Responses were observed after the administration of a single dose of avelumab to four patients, who also discontinued dosing due to severe irAEs. Patient 1 developed grade 3 CPK elevation and grade 1 transaminitis; patient 2 experienced elevated CPK and myositis; patient 3 developed the worst irAEs including grade 2 dysphagia, generalized muscle weakness, CPK elevation and transaminitis, and this patient was admitted to intense care unit; patient 4 developed grade 3 diarrhea. All patients recovered from these abnormalities after either oral or intravenous steroids treatment. The development of a response was accompanied by AEs, and this might be attributed to the disorder of immune homeostasis such as the TCR diversity change, which caused systemic influences. Despite these irAEs, no disease progression was observed for more than 14 weeks in responding patients. The main efficacy was confirmed to be derived from the blockade of PD-L1 signaling, and following the biopsy of two patients after treatment, the authors observed a replacement of thymocytes by activated CD8^+^ T cells. Additionally, investigators found that patients who responded to avelumab had been treated with the multikinase inhibitor sunitinib. All patients with irAEs had been treated with sunitinib, and two-thirds of patients who did not develop irAEs had never been treated with sunitinib. Sunitinib is an effective targeted drug in TET treatment [73], and it has also been confirmed as an immunomodulator that decreases the populations of Tregs and myeloid-derived suppressor cells [74, 75], possibly leading to the activation of autoimmunity. These findings suggest that sunitinib and other similar kinase inhibitors may alter the efficacy of ICIs and increase the risk of irAEs, but more evidence is needed.

## Nivolumab

Not all clinical trials acquired promising efficacy, a PRIMER single-arm, multicenter, phase II trial of 15 patients with unresectable or recurrent TC [64] reported that although treatment with nivolumab achieved a disease control rate of 73% (11/15), no significant tumor shrinkage was observed. Researchers suggested that further development or clinical trials of nivolumab were not recommended in these patients. Nevertheless, the results of this clinical trial were questionable since the sample size of this cohort was small, all patients with TC were from Japan, and the evaluation time was only 12 weeks. Therefore, clinical trials with larger sample sizes and longer evaluation times are needed to obtain more evidence for the efficacy of nivolumab in TET treatment.

## Ongoing clinical trials

More clinical trials with larger sample sizes are being conducted in patients with TETs (Table 3). Radiotherapy and/or chemotherapy may affect the in situ immune status and exert ectopic effects, which may improve the immunotherapy response and reduce adverse effects [76]. And the benefits of immunotherapy combined with radiotherapy or chemotherapy have been explored in many tumors, such as breast cancer, melanoma, and small cell lung cancer [77–79]. However, there is lack of such evidence in TET treatment. Some studies have documented the potential benefit of combined therapy, and several clinical trials are in progress. For instance, Yuki Katsuya et al. [51] observed a significant increase of PD-1/PD-L1 expression in 30 patients after chemotherapy, suggesting that chemotherapy combined with anti-PD-1/PD-L1 therapy or sequential therapy may provide greater therapeutic benefits. Different immune checkpoints have different functional mechanisms, and combined immunotherapy may produce more overall effects. Clinical trials simultaneously targeting PD-L1 and CTLA-4 have been conducted in patients with TETs. However, this combination may also lead to an increased frequency and severity of irAEs, as reported in clinical trials of anti-PD-1 (nivolumab) and anti-CTLA-4 (ipilimumab) combination therapy for melanoma [80]. In addition, clinical trials combining ICIs and specific targeted drugs such as epidermal growth factor receptor inhibitors, have also been registered. Notably, even for the combination of regimens with completely different functional mechanisms, the therapy-related adverse events may be more severe. For example, the combination of durvalumab and gefitinib results in high-grade liver enzyme elevations in 40–70% of patients, which is higher than that reported with either drug alone [81, 82]. These ongoing clinical trials will provide more information about the monotherapy or combination therapy using ICIs and facilitate the development of novel therapeutic strategies for the systemic treatment of patients with TETs.

### IrAEs of ICI therapy in patients with TETs

#### IrAEs reported in clinical trials and practice

**Fig. 4:**
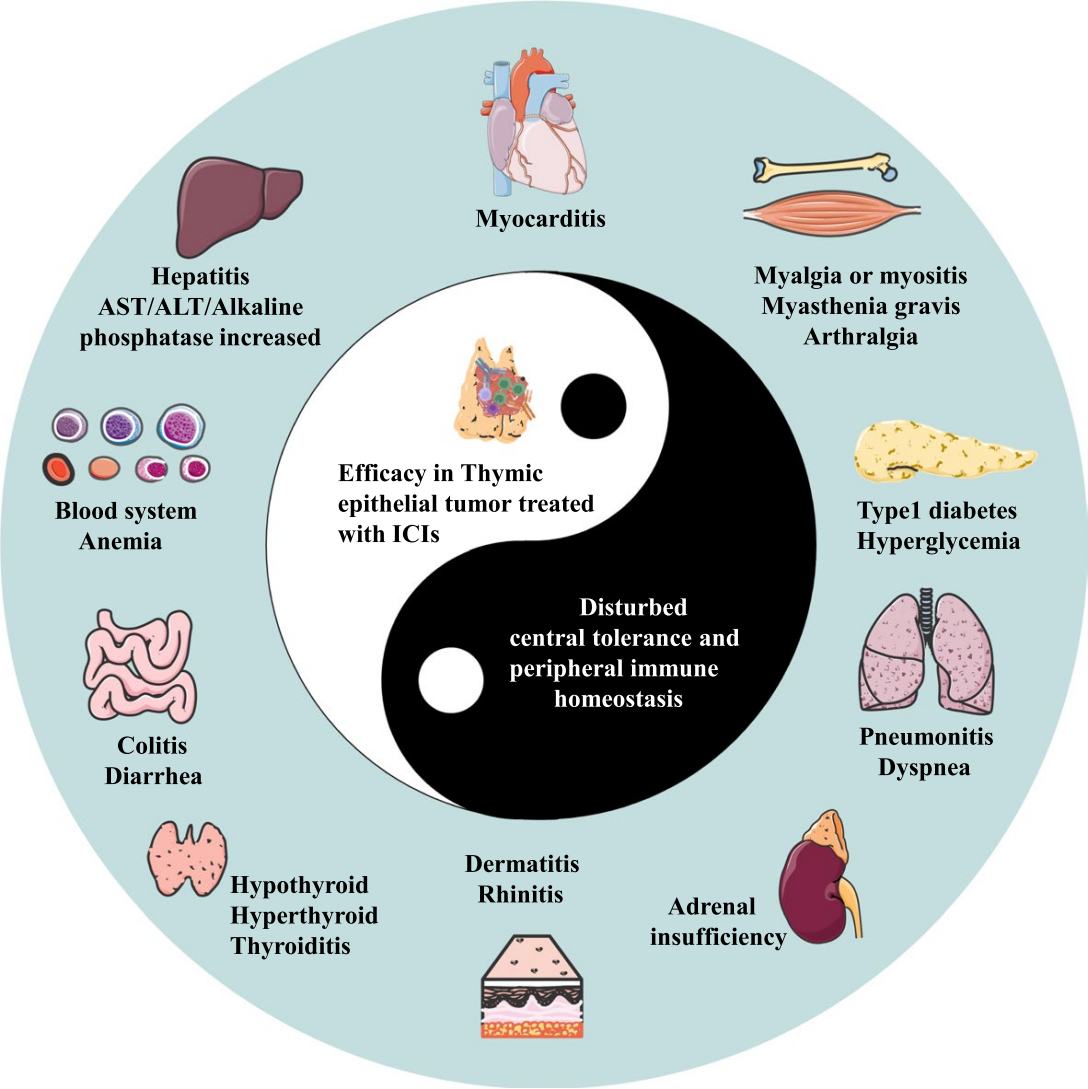
An overview of irAEs in patients with TETs receiving PD-1/PD-L1 inhibitors. Immunotherapy is systemically administered to affect not only tumor progression but also the whole immune system, including central tolerance in the thymus and peripheral immune homeostasis. irAEs: Immune-related adverse events; TETs: Thymic epithelial tumors

All clinical trials and most case reports in TETs immunotherapy reported irAEs of varying severity. They are very diverse and affect almost all organ systems (Fig. 4). Notably, irAEs seem more common in patients with thymoma. Cho et al. [62] reported irAEs in 15.4% of patients with TC compared with 71.6% of patients with thymoma. They suggested that immune checkpoint inhibitors should be avoided in patients with thymomas, and in patients with thymic carcinoma, immunotherapy should be considered with careful monitoring. All irAEs were graded according to the Common Terminology Criteria for Adverse Events, version 5.0 [83], listing them of different grades that occurred in the four completed clinical trials (Table 4). Patients with TETs usually have ADs or are in a preautoimmune state, and the irAEs caused by ICIs seem to be significantly higher than those in patients with other tumors [84, 85]. Although most patients only experienced mild irAEs (grade 1–2), a unique pattern of grade 3–4 irAEs was observed, including myocarditis, myositis and severe muscle weakness, which is rarely observed in patients with other tumors [61, 63, 85]. Remarkably, the development of irAEs appears to be associated with better therapeutic efficacy. Giaccone et al. [61] reported that four of nine patients (44.4%) who developed severe irAEs achieved partial responses, much higher than those without developing irAEs. Rajan et al. [63] also found that all responders developed irAEs, while only one of the four patients without response developed irAEs. Similar results have also been reported in patients with melanoma and NSCLC, with significantly higher response rates, PFS and OS in patients with irAEs than in those without irAEs [86–88]. In addition, some irAEs may occur several weeks after treatment, but the cumulative incidence of irAEs does not appear to increase with long-term follow-up [65], suggesting that a proportion of patients with TC are not at high risk of immune-mediated toxicity. Although irAE-related deaths are rare, further explorations of the mechanisms and more evidence that guide the identification of patients who might benefit from ICIs without developing severe irAEs are urgently needed [89].

**Table 4:** irAEs of different types and severity shown in completed clinical trials

Ref	Treatment	Patient(n)	irAEs		
			Grade I-II(No.)	Grade III(No.)	Grade IV(No.)
**Giaccone et al. **[61]	Pembrolizumab	40	AST increased (11) ALT increased (5) Alkaline phosphatase increased (10) Diarrhea (9) Arthralgia (4) Fever (5) Hypothyroidism (5) Rhinitis (4) Skin Rash (4)	AST increased (3) ALT increased (4) Dyspnea (3) Myalgia or myositis (3)	AST increased (2) ALT increased (1) Creatine phosphokinase Increased (2) Myocarditis (2) Hyperglycemia (1)
**Cho et al. **[62]	Pembrolizumab	33	Dyspnea (11) Chest wall pain (10) Anorexia (7) Fatigue (7) Cough (6) Myalgia (3) Anemia (2) MG (1)	Hepatitis (3) Anemia (1) MG (1) Thyroiditis (1)	Hepatitis (1) MG (1) Myocarditis (3)
**Rajan et al. **[63]	avelumab	8	Tumor pain (1) Extremity pain (1) Fever (2) Chills (1) Fatigue (4)	Autoimmune disorder (3)	Autoimmune disorders (2) Hypokalemia (1)
**Katsuya et al. **[64]	Nivolumab	15	Adrenal insufficiency (1) Rash maculopapular (4) AST increased (8) ALT increased (3) Hypothyroidism (1) Diarrhea (3) Creatine phosphokinase increased (3) Creatinine increased (3)	AST increased (1)	Not available

*irAEs* Immune related adverse events, *MG* Myasthenia gravis, *AST* Aspartate aminotransferase, *ALT* Alanine transaminase

### Molecular mechanisms of immunotherapy and irAEs

**Fig. 5:**
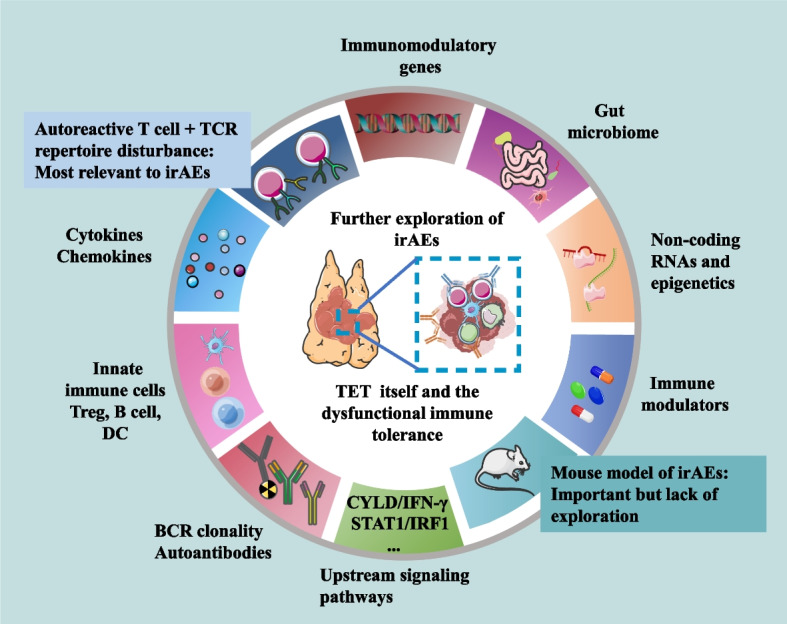
Some key issues requiring further mechanistic exploration. Comprehensive studies based on these issues will help researchers develop new biomarkers to prevent, monitor and manage irAEs during ICI therapy in patients with TETs. Autoimmune T cells and a disturbed TCR repertoire seem most relevant to irAEs, and a preclinical mouse model of irAEs is urgently needed to provide an ideal platform for mechanistic studies of irAEs. irAEs: Immune-related adverse events; ICIs: Immune checkpoint inhibitors; TETs: Thymic epithelial tumors

Although tumor immunotherapy is well-studied, little is known about the mechanisms of irAEs. Accumulating evidence indicates that some irAEs at least represent a decrease in self-tolerance mediated by abnormalities in T cells development, genetic susceptibility, B cells and other possible mechanisms (Fig. 5) [90]. Recently, Chen et al. [91] appealed more mechanistic studies on ICI resistance rather than performing additional clinical trials with combinations of different treatment schemes. Similarly, explorations aiming to elucidate the mechanisms of irAEs during ICI therapy for TETs should be strengthened before more clinical trials are conducted.

### Disturbed T cell development mediated by PD1/PD-L1 blockade

**Fig. 6:**
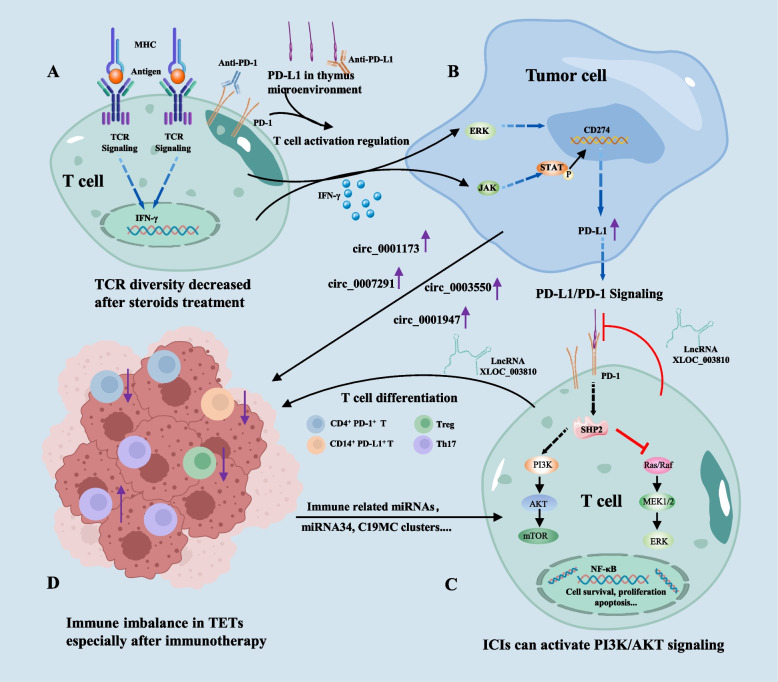
Cellular and molecular mechanisms of immune imbalance in TETs immunotherapy. **A**. PD-1/PD-L1 interaction in the thymic tissue regulates T cell development, and antigen-activated T cells upregulate PD-1 to avoid overactivation mediated tissue attack. **B**. Activated T cells release IFN-γ which upregulate PD-L1 expression in tumor cells via ERK/JAK2-STAT signaling pathways. Additionally, some circRNAs expressed by tumor cells may be involved in regulation of immune imbalance in TETs. **C**. Some lncRNAs, LncXLOC_003810, for example, can inhibit PD-1/PD-L1signaling and may be involved in regulating T cell differentiation. PD-1/PD-L1 signaling can inhibit PI3K/AKT pathway, which, however, can be activated by some immune related miRNAs. **D**. The immune microenvironment in TETs is distorted. PD-1/PD-L1 and their directly or indirectly interacting molecules play a vital role in maintaining immune homeostasis either inside or outside the thymus. However, this balance is disrupted in TETs especially after immunotherapy, which make TETs susceptible to irAEs during immunotherapy. TCR: T cell receptor; SHP2: Src homology 2 domain-containing tyrosine phosphatase 2; irAEs: Immune-related adverse events; ICIs: Immune checkpoint inhibitors; TETs: Thymic epithelial tumors

Systemic administration of ICIs inevitably affects T cells residing in normal thymic tissues, not only affecting central immune tolerance but also disrupting the homeostasis of the peripheral T cell receptor repertoire, leading to the production of autoreactive T cells. Firsly, thymic stromal cells and epithelial cells were confirmed to express PD-L1, especially in lymphocyte-rich thymoma, and the PD-1/PD-L1 interaction regulated both positive and negative selection of T cells in the thymus [47, 48]. Secondly, recent thymic emigrants in the peripheral blood are continuously activated via TCR signaling after encountering either autoantigen or alloantigen. And then, these activated naïve T cells will gradually express high-level PD-1 until they become exhausted T cells (Fig. 6 A). Under physiological conditions, normal function of PD-1/PD-L1 help establish immunologic homeostasis and protect normal tissues from attacked by exhausted T cells. However, blockade of PD-1/PD-L1 signaling may lead to disinhibition of effector T cells that induce thymic epithelial cell apoptosis and overcome either central immune tolerance to TSAs expressed by the thymic epithelium [92] or peripheral immune tolerance to circulating antigens. Moreover, PD-1 affects the CD8^+^ T cell status through intrinsic mechanisms such as functional inactivation or developmental regulation and promotes the differentiation of CD4^+^ T cells into Tregs [93]. In addition, the prognostic impact of the density and spatial architecture of tumor infiltrating lymphocytes was explored in TETs [94], and researchers demonstrated that high infiltration of stromal T helper and cytotoxic lymphocytes played a crucial role in anti-tumor immunity and might be potential marker predicting the efficacy of immunotherapy in TETs. The link between ADs and PD-1 has also been confirmed in a PD-1-deficient mouse model [95] that develops glomerulonephritis, inflammatory arthritis and lupus-like disease. In summary, blockade of PD-1/PD-L1 may impair their regulatory roles in both central and peripheral immune tolerance, thus leading to immune-related toxicities.

### Gene alterations and TCR diversity changes

Different haplotypes and polymorphisms of human leukocyte antigen in immune regulatory genes (such as CTLA-4 and PD-1) are associated with multiple ADs, and may play an important role in the development of irAEs [96–98]. Through gene sequencing analysis, researchers found enrichment of specific gene mutations in patients who developed irAEs compared to those without irAEs [99]. And significantly higher IFN-γ expression in T cells from patients who responded to pembrolizumab than in nonresponders was observed [100]. Notably, autoreactive T cells recognize autoantigens expressed by TET cells and release IFN-γ, and IFN-γ can upregulate PD-L1 expression via ERK/JAK2-STAT signaling pathways in tumor cells, which may be one of the reasons why patients with irAEs respond better to ICIs [101] (Fig. 6 A, B). These findings suggest that specific gene mutations may contribute to the development of irAEs and that TET mutational patterns should be considered when evaluating the risks of developing irAEs in patients receiving immunotherapy. Additionally, through TCR sequencing, Rajan et al. [63] found a trend in patients with irAEs toward a higher level of TCR diversity prior to therapy that decreased after treatment with steroids. The patient with the highest level of TCR diversity experienced the most severe irAEs. Läubli et al. [102] observed T cell infiltration in the irAE-related foci of patients treated with a PD-1 inhibitor, with characteristics similar to those of tumor-infiltrating T cells. TCR expansion may be a pharmacodynamic effect of ICIs, which reflects overall immune activation. However, the mobilization of abundant T cells and the increase in clonal diversity may lead to the production of autoreactive T cells and antibodies, increasing the risk of irAEs [103].

## B cells and antibodies

In NSCLC and renal cell carcinoma, researchers observed decreased numbers of circulating B cells and increased numbers of CD21^low^ B cells and plasmablasts after a course of ICI therapy. CD21^low^ B cells express higher levels of PD-1, and B cell receptor sequencing revealed greater clonality and a higher frequency of clones than CD21^high^ cells. Compared with patients with no B cell changes, patients with a 30% or greater reduction in the number of total circulating B cells and a two-fold or greater increase in the numbers of CD21^low^ B cells or plasmablasts were more sensitive to grade 3–4 irAEs [104–106]. Similar research in TET immunotherapy is also worth conducting because early changes in circulating B cells following immunotherapy may be biomarkers to identify patients who are at high risk of irAEs, and preemptive strategies targeting B cells may reduce toxicities in these patients. The analysis of baseline serum antibody profiles can also provide some insights into the mechanism of irAEs. Gowen et al. [107] identified a panel of specific antibodies that were differentially expressed in patients with severe irAEs. Autoantibodies against thyroid antigens or islet cell antigens were also detected in patients with thyroid dysfunction or diabetes after ICI treatment, but serological tests in patients with rheumatoid arthritis were usually negative [108–110].

### Immune-related epigenetic alterations in TETs

Large scale RNA sequencing data helps deeper understand the molecular mechanisms of TET development and their relationship with autoimmune diseases, which also provides insights to explore molecular mechanisms of TET immunotherapy and irAEs. Researchers identified some immune-related microRNAs (miRNAs) that are correlated with immune cell infiltration and type 2 macrophage polarization, some of which can regulate IFN-γor IL-10 signaling pathway to influence TETs microenvironment and PD-L1 expression [111]. They may also be involved in the pathogenesis of irAEs. For example, the downregulation of miRNA-146a was reported to be associated with an increased risk of irAEs [112]. In addition, miR-34 is confirmed to be highly expressed in early-stage thymomas but virtually absent in TC [50]. In NSCLC, P53 can downregulate PD-L1 via miR-34 [113], coincidently, TCs express PD-L1 more frequently than thymoma, and this may be caused by a decrease of miR-34. Except for non-clustered miRNAs, miRNA clusters-C19MC was also differentially expressed in thymoma and TC. In thymoma, miRNAs of C19MC are strongly expressed and can activate PI3K/AKT signaling pathway [114], which however, is inhibited by PD-1/PD-L1 axis [115], regulating TETs development and immune microenvironment (Fig. 6 B, C). Some other miRNAs were also reported to activate PI3K-AKT, FoxO, HIF-1 and Rap-1signaling pathways [116], indicating a synergistic role combining pathway inhibitors and immunotherapy, which will reduce the risk of irAEs. Considering the autoimmune correlation of thymoma rather than TC and the differential expression pattern of some miRNAs, miRNAs may be involved in autoimmunity related pathways in patients with thymoma. Although these miRNAs are absent in TC and patients with TC seldom suffer autoimmunity, the administration of ICIs can also activate autoimmunity related pathways. This may partially explain why patients with TC also appear irAEs after immunotherapy.

Moreover, researchers reported some differentially expressed circRNAs such as circ_0001173 and circ_0007291 in normal thymic tissue and TETs. Their upregulation in TETs was confirmed to be positively correlated with MAPK and TNF signaling, which mediate immune disorder in TETs and influence efficacy of immunotherapy [117]. Also, LncRNA XLOC_003810 was reported to regulate Th17/Treg balance in TETs with myasthenia gravis, and high expression of LncRNA XLOC_003810 results in a decrease of Treg cells and immune regulating factors [118], making patients more susceptible to irAEs. Interestingly, another study reported that LncRNA XLOC_003810 can promote the activation of T cells and inhibit the expression of PD-1/PD-L1, resulting in the upregulation of proinflammatory cytokines and a decrease of proportion of CD4^+^PD1^+^/CD4^+^PD-L1^+^ monocytes [119], influencing immunotherapeutic efficacy and the risk of irAEs (Fig. 6 B, D). These findings indicate that noncoding RNAs are potential biomarkers to predict and monitor immunotherapeutic efficacy and the occurrence of irAEs.

## Other potential mechanisms

Some cytokines, such as CXCL2/9/10 and IL-17, have been confirmed to be associated with irAEs induced by nivolumab in NSCLC or ipilimumab in melanoma. A higher level of IL-17 or a lower level of IL-6 correlates with a higher risk of developing irAEs [120–123]. Further investigations are also necessary to evaluate the roles of these cytokines in irAEs of TETs immunotherapy. The microbiota composition may be another indicator of irAEs in patients receiving ICI therapy. Routy et al. [124] showed that the effect of antibiotics on the gut microbiota is associated with adverse responses to PD-1 blockade, but the mechanism requires further investigation. In patients with melanoma, CTLA-4 inhibitor-induced irAEs were reported to be negatively associated with a high proportion of the *Bacteroidetes* phylum but positively associated with the *Faecalibacterium* genus and other *Firmicutes* species [99]. In patients with NSCLC, baseline enrichment of *Bifidobacterium* and *Desulfovibrio* in the gut microbiota was reported to be significantly associated with a lower incidence of irAEs during ICI therapy [125]. Similar research is needed for TETs to explore the relationship between the microbiota composition and the occurrence of irAEs.

### Strategies for the prevention and management of irAEs

Although ICIs improve the survival of patients with unresectable cancers, inevitable off-target conditions lead to autoimmune events at nontumor sites. Certain strategies and interventions may help prevent and manage irAEs that occur during TET immunotherapy. Rigorous evaluation of the indications for immunotherapy, especially the immune status of patients is needed since patients with immunity changes caused by any factors, such as aging, long-term immunomodulators and chronic virus infection, may be prone to side effects [90, 126, 127]. For patients with active immune instability or a history of ADs, immunotherapy is not recommended due to a lack of evidence from clinical trials. The administration of immunomodulators or immunosuppressants, such as steroids, concurrently with ICIs may be useful. Rajan et al. [63] reported that TCR diversity decreased after treatment with steroids in patients who developed irAEs. However, researchers have drawn inconsistent conclusions regarding whether the use of steroids to prevent or treat irAEs alters the efficacy of ICIs [128–130]. Furthermore, severe steroid-related toxicity was reported, including fatal infections [131]. Therefore, more research is needed to explore the effect of immunosuppressants on the efficacy of ICIs and to find their ideal regimen and timing. In addition, the dose of ICIs for TET immunotherapy must be optimized to minimize the risks of irAEs while maintaining efficacy. Although a higher incidence of irAEs was observed in patients treated with higher doses of ICIs [132], some studies reported that a lower dose of pembrolizumab also achieved significant efficacy with acceptable toxicity in patients with NSCLC and TETs [133, 134]. Importantly, irAES are variable and unpredictable that occur at the initial or later stage of treatment and even several months after the final course. Some patients experience a single irAE, while others may develop a series of irAEs simultaneously [135, 136].

Thus, a more reasonable approach seems to be closely monitoring irAEs via specific biomarkers and implementing timely measures to prevent their occurrence or minimize their risk. Radiomics provides comprehensive visualization and characterization of tissues of interest and associated microenvironments by automatically extracting high-fidelity, high-dimensional medical imaging features from standard images and has been shown to predict clinical outcomes, including irAEs, especially pneumonia [137]. Early changes in B cells may identify patients at high risk of irAEs, and strategies targeting B cells are worthy of development to reduce toxicity in these patients [105]. Cytokines, which are involved in the occurrence of irAEs, also serve as both predictive biomarkers and intervention targets [120, 121]. When irAEs occur, a timely severity assessment and the necessary management, such as oral or intravenous steroids, are needed. Discontinuation of immunotherapy is required for severe irAEs such as myocarditis and neuromuscular complications, and sometimes a gamma globulin infusion or plasma exchange should be performed.

## Readministration of ICIs in TETs

The readministration of ICIs after initial irAEs is challenging and controversial, which requires a careful assessment of the risk and potential clinical benefits. Evidence from case reports is insufficient [101, 102], and rigorous clinical research with a large sample size is needed, particularly to evaluate the ICI dose correlation with the occurrence of second-time irAEs. The antitumor activity and tolerability of readministering the same ICI at different doses was explored in other tumors, including NSCLC, renal cancer, and melanoma. Considering different immunological mechanisms of different ICIs, switching to another ICI after irAEs occur has also been evaluated, and readministration of ICIs appears feasible for low-grade irAEs [138–144]. A study examined the effect of a PD-1 inhibitor on patients with melanoma who had experienced ipilimumab (CTLA-4 inhibitor)-associated immune toxicity and found that most irAEs observed were new rather than recurrent [129]. Clinical trials in patients with NSCLC and melanoma have reported a dose correlation between the second-time irAEs with CTLA4 inhibitor, but not PD-1/PD-L1 inhibitor. And an adjustment of drug dose or course interval was not recommended for PD-1/PD-L1 inhibitor, but required for CTLA4 inhibitor after the initial irAEs [145, 146]. In addition, concurrent immunosuppressors or immunomodulators, such as vedolizumab (an integrin inhibitor) and TNF-α inhibitors, were administered to decrease the risk of re-emergence of irAEs, and a lower risk of second-time irAEs was confirmed than in patients who were administered ICIs alone [141, 147]. In patients with TETs, concurrent use of cyclosporine A with avelumab was also reported to prevent the development of second-time irAEs in patients with previous immune-mediated myositis [63]. These findings indicate that readministration of ICI with either the same/different doses or different types is feasible in carefully selected patients after balancing risks and benefits. However, for patients who have experienced life-threatening immunotoxicity, reintroduction of ICIs may not be reasonable, and further research is needed to accumulate more evidence.

## Insights and future perspectives on ICIs in TETs

PD-1/PD-L1-based ICIs show promising prospects in TET treatment, but high-frequency irAEs pose a challenge. Several issues merit further study to maximize the therapeutic benefits while minimizing the risks of irAEs. First, new biomarkers are urgently needed to screen patients who might experience potential benefits. The main criterion for evaluating patients who might benefit from ICIs is PD-1/PD-L1 expression levels, but they are not perfect biomarkers [148] and do not reflect the propensity to develop irAEs. Second, clinical trials of patients with unstable immune status should be conducted. Patients with ADs are usually excluded from clinical trials of ICIs for tumors, which is particularly restrictive for patients with TETs [61–63], resulting in a serious lack of evidence for the guidance of immunotherapy. Clinical trials of ICIs in patients with melanoma and NSCLC presenting with ADs have reported mild irAEs and similar response rates to those without ADs, and irAEs usually do not lead to a discontinuation of ICIs [128]. Therefore, clinical trials of ICIs for patients with TETs complicated with ADs are also needed to accumulate more evidence for medication. Third, changes in some biomarkers related to irAEs are noted, which are presumed to be involved in the mechanisms of irAE occurrence and have the potential to serve as monitoring factors [90, 105, 121]. Nevertheless, much more research is needed before the clinical application of these biomarkers. A study combining radiotherapy and immunotherapy was conducted in mouse models of subcutaneous in situ thymomas to study the changes in cytokine levels in the tumor immune microenvironment and explore the effect of combined therapy [149]. However, no preclinical mouse models that mimic the autoimmune and toxic events observed in patients have been developed to understand the biological mechanisms of irAEs [150]. Fourth, standardization of the management of irAEs of different types and severity, assessments of the criteria for readministration of ICIs after initial irAEs, and recommendations for the time interval and corresponding precautions are needed. With an increasing number of clinicians trying to use individual ICIs, the standardized guiding framework will help to improve the efficacy of ICIs in patients with TETs and reduce the occurrence of severe irAEs. In addition, many researchers focus on irAEs, but mechanisms of nonresponse and resistance are also needed. Some potential mechanisms have been reported, such as defects in class I antigen presentation, defects in the Wnt/β-catenin and interferon signaling pathways [151], and overexpression of alternative immune checkpoints, such as T cell immunoglobulin and mucin domain-containing molecule-3 [152]. In all, more explorations of irAE mechanisms combined with evidence from clinical trials will increase the prospects of immunotherapy especially ICIs for patients with TETs.

In conclusion, although ICI therapy is at the early stage of TETs treatment, it has been proven the ability to induce durable response in a subset of patients with TETs. However, the risk of developing life-threatening irAEs hampers their adoption. An understanding of the mechanisms of irAEs, the identification of predictive biomarkers, and development of risk countermeasures to make immunotherapy a safe and effective regimen are urgently needed, which will improve survival and the quality of life of patients with TETs.

## Data Availability

Not applicable.

## Abbreviations

PD1: Programmed death receptor 1
PD-L1: Programmed death receptor 1 ligand 1
TETs: Thymic epithelial tumors
ICIs: Immune checkpoint inhibitors
irAEs: Immune-related adverse events
ADs: Autoimmune diseases
CTLA-4: Cytotoxic T lymphocyte-associated protein 4
NSCLC: Non-small cell lung cancer
OS: Overall survival
PFS: Progression-free survival
